# Microbial Diversity in Extreme Marine Habitats and Their Biomolecules

**DOI:** 10.3390/microorganisms5020025

**Published:** 2017-05-16

**Authors:** Annarita Poli, Ilaria Finore, Ida Romano, Alessia Gioiello, Licia Lama, Barbara Nicolaus

**Affiliations:** Consiglio Nazionale delle Ricerche (C.N.R.), Institute of Biomolecular Chemistry (I.C.B.), Via Campi Flegrei 34, 80078 Pozzuoli, (Na), Italy; apoli@icb.cnr.it (A.P.); ifinore@icb.cnr.it (I.F.); iromano@icb.cnr.it (I.R.); alessia.gioiello@unina.it (A.G.); llama@icb.cnr.it (L.L.)

**Keywords:** biodiversity, extreme habitats, extremophiles, enzymes, polymers, osmolytes

## Abstract

Extreme marine environments have been the subject of many studies and scientific publications. For many years, these environmental niches, which are characterized by high or low temperatures, high-pressure, low pH, high salt concentrations and also two or more extreme parameters in combination, have been thought to be incompatible to any life forms. Thanks to new technologies such as metagenomics, it is now possible to detect life in most extreme environments. Starting from the discovery of deep sea hydrothermal vents up to the study of marine biodiversity, new microorganisms have been identified, and their potential uses in several applied fields have been outlined. Thermophile, halophile, alkalophile, psychrophile, piezophile and polyextremophile microorganisms have been isolated from these marine environments; they proliferate thanks to adaptation strategies involving diverse cellular metabolic mechanisms. Therefore, a vast number of new biomolecules such as enzymes, polymers and osmolytes from the inhabitant microbial community of the sea have been studied, and there is a growing interest in the potential returns of several industrial production processes concerning the pharmaceutical, medical, environmental and food fields.

## 1. Introduction

**Figure 1:**
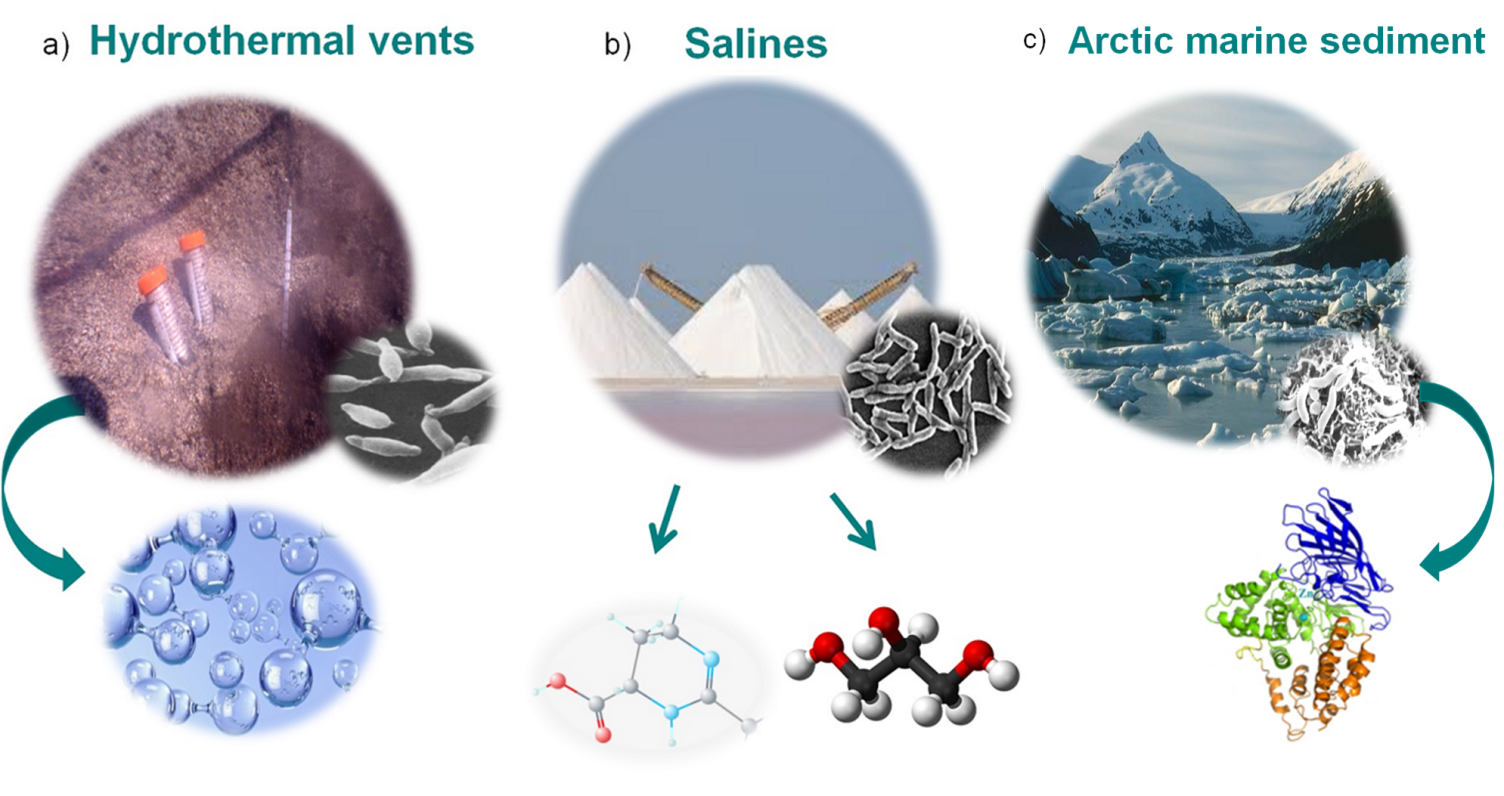
Example of extreme aquatic habitats and the extremophiles and the molecules they produce. (**a**) Hydrothermal vents: The hyperthermophilic *Thermotoga neapolitana*, a hydrogen-producing bacterium isolated at “Secca Fumosa” in the Gulf of Pozzuoli, Naples, Italy. (**b**) Salines: The halophilic *Chromohalobacter salexigens*, a bacterium isolated at the island of Bonaire, Netherlands Antilles. The osmoadaptation is achieved by the accumulation of several types of osmolytes such as ectoine and glycerols. (**c**) Arctic marine sediment: The marine psycrophile *Colwellia psychrerythraea*, producing a cold-active aminopeptidase (ColAP), isolated from Arctic marine sediments.

Ecosystem biodiversity has become an object of intensive study; consequently, a great deal of information has been gathered on the distribution of microorganisms around the world. In addition, there is growing interest in the role of marine microorganisms in biogeochemical processes, biotechnology, pollution and health. In recent years, many authors have focused on the great potential of marine microbes for use as prolific producers of bioactive substances, and they have exploited the vast marine microbial treasures for utilization as sources of drugs and antimicrobial agents [1,2,3,4,5,6,7,8,9,10,11,12,13,14,15,16,17,18]. Extremophiles are organisms that are capable of living in particular ecosystems that are characterized by high (Thermophiles) or low temperatures (Psycrophiles), high ionic strength (Halophiles), acidic or alkaline pH values (Acidophiles, Alkalophiles), anaerobic conditions (Anaerobe), high pressures (Piezophiles), UV radiations and Polyextremophiles e.g., Thermoacidophiles and Thermohalophiles. Moreover, in some particular habitats such as the deep-sea piezosphere, in which the pressure could be greater than 10 MPa (or greater than 1000 m in depth), the temperature can be cold (2–3 °C) or can be very hot (up to 400 °C or higher for example near the hydrothermal vents) and subsequently, psychro-piezophilic or thermo-piezophilic microorganisms, can be found, respectively [2,3]. In fact, marine extremophiles grow in hot, cold and salty environments; therefore, it is expected that their lipids, enzymes and biopolymers will present unique properties for adapting to extreme conditions (Figure 1) [2,3,4,5,6,7].

The existence of life at high temperatures has raised important questions about the molecular mechanisms that extremophiles have developed to make it possible for them to grow at temperatures well above the boiling point of water. The strategy for biomolecule stabilization in response to thermal stress is multifactorial, and it involves the investment of all the cellular components such as DNA, RNA, proteins, ribosomes, cell membranes, and biopolymers [2,8,9,10,11,12]. Particular attention has been concentrated on thermophilic microorganisms from marine hot springs and hydrothermal vents because of the marked ability of some of them to produce and secrete polymers and enzymes that are suitable for industrial purposes [12].

Discovery of hyperthermophilic archaea have helped to redraw the evolutionary tree of life, forming the third evolutionary branch [8]. These origins were sought in particular ecosystems that closely replicate the environmental characteristics that were present on Earth millions of years ago. Within this scope, it is believed that in the dark and cold deep sea, the hydrothermal vents represent the only systems in which primitive life was protected from the environmental changes that occurred on our planet [13,14,15].

Conversely, there are the cold environments that are conventionally considered to be inhospitable because of their physical and chemical properties, and they have always been defined as extremes that are believed to be scarcely compatible with life. The literature concerning microbial life at low temperatures is relatively recent, and at present, it is widely believed that several forms of life, both prokaryotes and eukaryotes, proliferate in these chilly places thank to a series of adaptations that allow them to carry out every vital function [13,14,15,16].

In addition, microorganisms that inhabit extreme saline habitats have been considered useful objects for ecological and evolutionary studies. One reason for this interest is the need to understand the biochemical mechanisms involved under these extreme conditions because of possible biotechnological uses for their enzymes and molecules such as exopolysaccharides, polyhydroxyalkanoates, osmolytes, carotenoid pigments, bacteriorhodopsin, etc. [11,17].

The first studies on extremophiles from extreme habitats were based on strain isolation that employed classic culture-dependent approaches. With this method, only microorganisms whose metabolic and physiological requirements can be duplicated in the laboratory, could be isolated. To overcome this limitation, metagenomic approaches have recently been developed to explore and access the uncultured microbial community [18,19,20].

More and more marine microorganisms, particularly extremophile microbes from marine hot springs and hydrothermal vents, are attracting attention for their marked ability to produce enzymes, polymers, osmolytes, etc.

Marine environments represent the richest source of new genes, enzymes and natural products for use in industrial processes. In particular, the microbial species that live under extreme conditions are an extraordinary natural source of stable and efficient enzymes, which could improve the performance and sustainability of industrial processes by making new processes available for the future, making applications and biotech products more economical and ecological [19,20,21,22,23]. The development of more automated and affordable techniques for isolating and characterizing marine microbial bioactive metabolites would make natural product extracts from marine microbes more accessible [18].

The present review is part of a large-scale investigation that many researchers have performed to analyse the biodiversity of marine extremophiles and aims to focus mainly on microbes that are directly linked to production of biomolecules.

## 2. Habitat: Hydrothermal Vents

Deep-sea hydrothermal vents are unique environments that provide the partial or complete energy/nutrient fluxes necessary to supporting the diverse microbial communities that are distributed along the temperature range, and reduced compound gradients are more or less correlated with the transition from anoxic to oxic conditions [24]. The large population of animals that surround the volcanically-driven warm vents (primarily tubeworm communities and an array of crabs, shrimp, giant clams and gastropods) is supported by the growth of chemoautotrophic microorganisms [25]. These microorganisms are the bases of the hydrothermal trophic food chain, and they can exist as free-living organisms that are associated with the vent fluids that are likely growing and reproducing within the sub-seabed system. They are free-living microbial carpets growing on the surface that is exposed to flowing vent waters or endo- and exosymbiotic associations with invertebrates and within the deep sea hydrothermal vent plumes. Here, the most frequently studied physical parameter that limits microbial life is the temperature; in fact, *Bacteria* and *Archaea* from hydrothermal vents have been extensively investigated in smoker fluids, black smoker sulphides and sediments that have high temperatures, for the purpose of hypothesizing and also speculating on the origin of life [26].

### 2.1. Microbial Diversity

#### 2.1.1. Hyper-Thermophilic and Thermophilic Microorganisms

The primary microbial species that have been isolated and are growing under strict anaerobic, extreme temperature and pressure conditions belong to *Archaea* kingdom, namely Euryarchaeota and Crenarchaeota [27]. Euryarchaeota include thermophiles (*Pyrococcus* and *Thermococcus*), methanogens (*Methanococcus* and *Methanopyrus*) and sulphate and iron reducers (*Archaeoglobus*) organotrophs, nitrate reducers, sulfur reducers and aerobes, whereas Crenarchaeota includes thermophilic and hyperthermophilic heterotrophs (*Hyperthermus*, *Staphylothermus* and *Desulfurococcus*) (Table 1). Most of the heterotrophic species exhibit maximal growth temperatures of less than 105 °C, while *Pyrolobus fumarii* at 113 °C and strain 121, a member of the *Desulfurococcales*, at 121 °C [28]; *Pyrodictium* and the methanogen *Methanopyrus* species grow at 110 °C and 122 °C [29]; the latter is the highest known temperature at which it is possible to grow a pure culture.

#### 2.1.2. Thermopiezophilic Microorganisms

Several thermophilic or hypertermophilic microorganism are known to be piezophilic. Some piezophiles are able to grow at or slightly higher than atmospheric pressure (0.1–10 MPa) and are considered to be piezotolerant. Others are classified as piezophilic or hyperpiezophilic, which require high pressure for optimal growth 10–50 MPa and >50 MPa, respectively, for piezophiles and hyperpiezophiles. Hyperpiezophiles that grow at pressures as high as 130 MPa have been isolated [2]. This means that of course in the laboratory, special high-pressure equipments, such as pressure vessels, hydrostatic pumps, etc., are necessary in order to study piezophiles, from sampling of deep-sea sediments or seawater to isolation [3]. *Thermococcus* species are the predominant group from hydrothermal areas and can also host piezophiles that can be found in various pressure and temperature regimes. *Methanococcus jannaschii* isolated at a depth of 2600 m growth under hyperbaric conditions of about 75 MPa. Alain et al. [34] first reported the isolation of a thermopiezophilic sulfate-reducing bacterium, *Marinitoga piezophila* strain KA3T, from the East-Pacific Rise at 2630 m. Its optimal growth temperature and pressure were 65 °C and 40 MPa, respectively. Recently, two novel thermo-piezophilic, chemolithoautotrophic Proteobacteria from the Mid-Atlantic Ridge were isolated: *Thioprofundum lithotrophicum* strain 106, phylogenetically associated with many of the thiotrophic endosymbionts found in deep-sea (mussels, clams and tubeworms) in the class Gammaproteobacteria, and *Piezobacter thermophilus* strain 108, phylogenetically affiliated with the family Rhodobacteraceae (Alphaproteobacteria class) [35].

#### 2.1.3. Some Examples of Hyperthermophiles, Thermophiles and Mesophiles from Different Marine Areas

The Lost City, hydrothermal vents, are different from black smokers as they are characteristically contain calcium carbonate and magnesium hydroxide minerals and rarely contain volcanic rocks; they are constituted of chimneys 30–60 m tall localized at water depths of 750–870 m. The fluids venting chimneys range in temperature from 40–90 °C and are highly alkaline (pH 9–11), with high a concentration of dissolved H_2_, CH_4_, low molecular weight hydrocarbons but almost no dissolved CO_2_ [36].

The porous walls of structures in hydrothermal areas host hyperthermophiles, thermophiles and mesophiles. A methane-metabolizing *Archaea* related to the *Methanosarcinales* that grows at 80 °C is the predominant group that thrives in these edifices, forming biofilms that are approximately 10-cm thick that adjoin to the hydrothermal flow. The dominant genera, *Thermotoga* and *Desulfurobacterium*, are sulphur-reducing, thermophilic, anaerobic and they grow over a temperature range from 60–80 °C. *Thermus* and *Bacillus* are thermophilic, heterotrophic and aerobic, and they grow over a range from 60–75 °C. Representative species that have been assigned to the *Thermotogales* order are *Thermotoga maritima*, *Thermotoga neapolitana*, and to *Firmicutes* order are *Thermoanaerobacter ethanolicus* and *Thermosipho melanesiensis*.

Shallow water submarine hydrothermal vents represent a natural system different to that described above for the diverse pressure and temperature values, but they are more easily accessible [37].

The coastal zones of the southern Tyrrhenian Sea (Flegrean area, Cape Palinuro, Eolian Islands) represent a vent that is easily accessible by diving [37]. An analysis based on the Fluorescent In Situ Hybridization molecular and Denaturing Gradient Gel Electrophoresis (DGGE) technique found that, although further investigations are needed to elucidate the relationship between *Archaea* and *Bacteria* and their relative abundances at shallow vents, the predominance of *Bacteria* over *Archaea* currently represents the major difference between shallow and deep-sea hydrothermal vents.

*Pyrococcus*, *Thermotoga*, *Thermococcus*, *Archeoaglobus*, *Methanococcus*, *Pyrodictium*, *Aquifex* and *Igneococcus* thrive in both shallow water and deep-sea hydrothermal systems. Members of *Thermococcus* (*T. celer* and *T. litoralis*) have been isolated from coastal hydrothermal systems [38]. These bacterial communities have likely been sustained for long (geological) periods of time, given the stable hydrothermal activity at sea vent sites (Table 2).

Other examples of thermophilic microorganisms that have been isolated from hot environments and the ability to produce biomolecules are reported in Table 3.

### 2.2. Metagenomic Analyses

To attain a more complete microbial taxonomic description, different approaches have been utilized; the use of molecular biology techniques has identified a high level of microbial diversity, including numerous uncultivated species that are representative of this unique ecosystem [52]. An analysis of thin petrographic cross-sections from the outside to the inside of the chimney spires (white smokers) and 16S rRNA gene cloning have revealed distinct microhabitats for different bacteria and archaea [63,64,65,66,67,68,69].

In addition, the beginning of the metagenome sequencing era has opened up new horizons in understanding the complexity and versatility of extremophiles. For example, in (hyper)thermophiles, sulphur-cycling genes are predominant in the species that inhabit sulphidic-deep sea hydrothermal vents that are characterized by very high temperatures, while microorganisms that live in serpentinite hydrothermal vents possess genes for H_2_ oxidation.

A metagenomic study conducted on deep-sea hydrothermal vent chimneys harboured in the Mothra at the Juan de Fuca Ridge revealed that the metagenome was rich in genes involved in the DNA repair system and homologous recombination, suggesting that strategies were developed for extreme condition survival. In addition, genes for chemotaxis and flagellar motion were highly enriched in the chimney metagenomes, reflecting a dynamic situation that was present within the chimney walls [70]. Similar results were found in another metagenomic study carried out in an extreme acidic environment, it was also enriched in genes coding for DNA and RNA repair system and chaperons, which gives supports to this hypothesis [71]. The microbial communities in venting sediments from the Jan Mayen vent fields in the Norwegian-Greenland Sea have been analysed by using different approaches such as metatranscriptomics, metaproteomics and metagenomics. These sediments hosted communities of *Epsilonproteobacteria*, *Deltaproteobacteria* and *Gammaproteobacteria*, aside from archaeal taxa, ciliates and nematodes. From a metabolic point of view, these communities activated sulphur and methane oxidation genes, carbon fixation pathways and aerobic and anaerobic respiratory chain genes [72].

### 2.3. Applications

#### 2.3.1. Thermophilic Microorganisms and Their Metabolic Peculiarity

The great amount of interest regarding thermophilic *Bacteria* and *Archaea* is based on the investigation of molecular mechanisms that have allowed for the thermal adaptation of cellular components such as lipids, proteins and nucleic acids. The composition of archeal lipids differs from bacterial types in terms of linkages via ether bonds between phytanyl chains to glycerol or to other alcohols. Bacterial lipids have linked glycerol via ester bonds to two fatty acids acyl chains, and they are organized in a bilayer, whereas in extreme thermophilic and acidophilic archaea, tetraether lipids spanning the entire membrane to form a monolayer have the thickness of a regular lipid bilayer [73] The tetraether lipid structures of the hyperthermophilic *Archaea* increase their cyclization of C40 isoprenoid chains upon heat stress; this response allows for a decrease in the motion of lipids and contributes to acceptable membrane fluidity at elevated temperatures [74].

#### 2.3.2. Thermozymes and Biopolymers

The use of metagenomic sequence-based screening represents an easier way to provide new thermozymes from thermophilic and hyperthermophilic microorganisms. Polysaccharide-degrading enzymes, such as amylases, (hemi)cellulases, chitinases and pectinases but also thermozymes with lipolytic and proteolytic activities, are required for industrial purposes as well as in biorefineries (Table 3) [23,45,75,76].

Extremophilic microorganisms from shallow and deep-sea hydrothermal vents and their extraordinary diversity represent great potential also for biotechnological applications of their exopolysaccharides [10,11,19,39,77,78]. An example of the recent implications of biomolecules for thermophile applications relies on the opportunity to reuse vegetable waste materials as a source of value-added bioproducts, which is performed by using thermostable enzyme activities (Table 3) [79,80].

Several interesting examples of biologically and biotechnologically useful exopolysaccharides (EPSs) from extremophilic microorganisms have been described recently. The EPSs of two marine bacteria isolated from a deep-sea hydrothermal vent, namely *Vibrio diabolicus* and *Alteromonas infernus*, have been investigated for their potential applications in regenerative medicine [62]. In particular, the EPS (HE800) from *V. diabolicus*, is commercialised under the name Hyalurift^®^, is a linear hyaluronic acid-like polymer (MW 8 × 10^5^ Da) that stimulates collagen structuring and extracellular matrix settling in dermal fibroblasts. Because of these properties, it has been investigated for its regenerating activity in bone and skin. The EPS (GY785) from *A. infernus* is a branched and low-sulphated polysaccharide (MW ~10^6^ Da) that is able to improve the mechanical properties of the cellulose-based hydrogels used for cartilage tissue engineering applications. Moreover, following free radical depolymerisation and sulphation, these two polymers are able to inhibit complement activity [81]. These results are very important for treating diseases caused by alterations in the immune system and the hyper-activation of the complement system.

Two EPS-producing strains, *Bacillus thermodenitrificans* strain B3-72 and *B. licheniformis* strain B3-15, have interesting anti-viral activities and were isolated from the hot shallow marine vents of Vulcano island (Italy) [21]. Moreover, another strain of *B. licheniformis*, strain T4, was isolated from Panarea island (Italy), and it produced a fructo-glucan EPS with anti-cytotoxic activity (Table 3) [78].

#### 2.3.3. Other Compounds from Marine Thermophiles

Halophilic or halotolerant bacteria and eukaryotes tend to accumulate neutral osmolytes, whereas halophilic or halotolerant archaea, hyperthermophilic archaea and thermophilic bacteria tend to accumulate negatively charged solutes due to their carboxylate or phosphate groups [82].

In thermophilic and hyperthermophilic microorganisms, mannosylglycerate, mannosylglyceramide, di-glycerol-phosphate, and α-glutamate are accumulated primarily in response to salt stress, whereas di-myo-inositol-1,1’-phosphate (DIP) and cyclic-2,3-bisphosphoglycerate increase primarily in response to high temperatures. In fact, a stronger accumulation of DIP is observed in *Thermotoga maritima*, *Thermotoga neapolitana* and *Pyrococcus furiosus* (101 °C), in particular in the last named species, 20-fold increase in DIP. Gonçalves et al. [83] reported the consecutive actions of di-myo-inositol phosphate synthase and inositol-1-phosphate cytidylyltransferase in the synthesis of DIP and the chance that the early ancestor for DIP synthesis thrived in a marine-like environment.

The thermohalophilic archaeon *Thermococcus litoralis* accumulated mannosylglycerate, aspartate, α-glutamate, DIP, hydroxyproline, trehalose and beta-galactopyranosyl-5-hydroxylysine; these latter three compounds were not detected in most of the *Thermococcus* genera. The novel compatible solute 1-glyceryl-1-myo-inosityl phosphate was found in the microaerophilic thermohalophile *Aquifex pyrophilus* [84]. The presence of trehalose (a non-reducing glucose disaccharide) in the marine species *Pyrobaculum aerophilum* is very interesting and indicates that this solute can also be used as an osmolyte in organisms that grow at extremely high temperatures, and not only as a cryoprotectant for the freeze-drying of biomolecules.

α-Glutamate (α-aminoglutaric acid) represented 37% of the total osmolytes isolated from the anaerobic thermohalophile *Methanothermococcus thermolithotrophicus*, and this compound is present as a major component of anoxic sea sediments [85].

## 3. Habitat: Cold Marine Environments

When speaking of “cold marine environments”, our mind immediately thinks of the two geographical areas located at the extremities of our planet, the north Arctic continent and south Antarctic continent. Indeed, when it changes as a function of the latitude and the seasons, the temperature of marine water decreases with the depth. In particular, under the thermocline (the body of water in which the temperature changes rapidly with the depth), the temperature ranges from 4–5 °C or lower. Furthermore, considering that the terrestrial crust is covered by seas over approximately 70% of its surface, and, of this, 90% is marine water reaching 5 °C or less, and that approximately 15% of the world's oceans are covered by sea ice during part of the year, Earth can be considered a “cold marine planet” [86].

In particular, cold-loving and cold-tolerant microorganisms are found among the psychrophiles and psychrotrophs, according to their growth temperature. In particular, lower temperatures are preferred by the so-called psychrophiles or microorganisms that proliferate between 0 and 20 °C, with an optimum at 15 °C or less, while psychrotrophs are defined as microorganisms that are able to tolerate cold temperatures and have an optimal growth temperature at 20 °C. Many of the cultured psychrophilic are also piezophiles and belong to the Gammaproteobacteria class and are species from one of five main genera: *Shewanella*, *Photobacterium*, *Colwellia*, *Moritella* and *Psychromonas* [2].

It is worth noting that in cold marine waters, the waters are denser as the temperatures decrease, and therefore, energy and nutrient diffusion slows down. These environmental conditions suggest that psychrophilic microorganisms can be oligotrophic (essentially, oligocarbophiles), meaning that their metabolism is adapted to low nutrient concentrations.

A recently investigated cold niche is represented by water drops entrapped in the middle of sea-ice crystals. There, water veins reach temperatures lower than 5 °C. This cold environment is characterized by the presence of water and ice, and it hosts microbial life forms that were named for the first time as eutectophiles by Deming [87]. This result is particularly interesting because beyond low temperatures, there is a high solute concentration at the interface between sea-ice crystals and marine water that permits a high rate of microbial uptake. Here, psychrophilic microorganisms can experience eutrophic conditions, and therefore, they could be related to halophiles, in the case of higher salt concentrations [88]. Given the nature of the environments that are colonized by psychrophiles, they are often able to thrive in the presence of more than one stress factor.

### 3.1. Microbial Diversity

In Table 4 selected psychrophilic microorganisms isolated all over the world are reported, from the Arctic to Antarctic, from the Red Sea to the South of China, from the deep-sediment of the Japan Trench to the Pacific Ocean. The temperature of isolation sites ranged from −2 °C to 28 °C.

The ability to produce biomolecules from marine psycrhophilic microorganisms are reported in Table 5.

#### 3.1.1. Psychrophilic Microorganisms

Taxonomically, cold-loving and cold tolerant microbes are found in both *Archaea* and *Bacteria* domains and they are distributed over numerous genera, such as *Arthrobacter*, *Colwellia*, *Exiguobacterium* [111], *Gelidibacter*, *Glaciecola* [112], *Halobacillus*, *Halomonas* [113], *Hyphomonas*, *Listeria* [114], *Marinobacter* [115], *Methanococcoides*, *Methanogenium* [116], *Moritella* [117], *Planococcus* [118], *Pseudoalteromonas*, *Pseudomonas* [119], *Psychrobacter*, *Psychroflexus* [120], *Psychromonas* [104], *Psychroserpens* [100], *Shewanella* [121] and *Sphingomonas* (Table 4).

#### 3.1.2. Adaptation Strategies of Psycrophilic Microorganism and Their Metabolic Studies

They can be Gram-positive and Gram-negative, autotrophic and heterotrophic, aerobic and anaerobic, phototrophic and non-phototrophic. These microorganisms are all equipped with cold-adapted cellular mechanisms that make them suitable inhabitants for environments that are characterized by very low temperatures, and, for this reason, they are attractive for scientific investigation. It has become apparent that, first of all, the “interface” between the microbe and the surrounding cold environment is adapted; as the temperature decreases and the flexibility of the cell membrane changes, guaranteeing the fundamental role of nutrient uptake and intracellular osmoregulation.

In fact, the membrane lipid compositions of psycrophilic cells present un-saturated, poly-unsaturated, short, branched and cyclic fatty acids with a higher percentage with respect to mesophilic and thermophilic microorganisms. Bowman et al. [105] reported the fatty acid composition of the psychrophilics *Shewanella gelidimarina* and *Shewanella frigidimarina*, both of which were isolated in Antarctica and produce eicosapentaenoic acid (20:5 omega 3). In addition, for many species belonging to the *Colwellia*, *Moritella*, *Photobacterium*, *Psychromonas*, and *Marinomonas* genera, the presence of insaturations in the lipid composition was revealed [16,122]. Un-saturated diether lipids have been detected in the psychrophilic archaeon *Halorubrum lacusprofundi* [123]. In addition, it was observed that the amount of un-saturations on the lipid chains increases as the temperature decreases, generation after generation.

Other cellular adaptation mechanisms concern the stability of the single-stranded RNA conformation, when it is subjected to a low temperature. Most of the investigated psychrophiles present small proteins bound to the RNA; they are known as “cold shock proteins” (Csp), and they guarantee the permanence of the RNA structure [124,125,126,127,128]. Genomic studies of psycrophilic microorganisms have shown the presence of csp genes, in variable numbers, from 12 cps genes in the genome of *Psychromonas ingrahamii* 37, which was isolated from a sea ice sample collected in northern Alaska [125].

### 3.2. Metagenomic Analysis

Recent studies that were conducted on aquatic psychrophilic populations revealed a trophic complexity and diversity of communities that was much higher than expected. More than 40% of the sequenced psychrophiles have been isolated from marine environments (from the Pacific Ocean and the Southern Ocean all around Antarctica). For example, the cold oligotrophic desert soils of the Antarctic continent are widely considered to be one of the extreme environments on earth and recent studies have highlighted that in general microbial biomass is orders of magnitude higher than originally thought and the microbial community complexity and diversity is much higher than predicted exhibiting surprisingly rapid structural changes in response to changing environmental conditions. The Antarctic continent harbours unique niches, such as the sub-glacial ice habitats. Analyses of the former have revealed a community dominated by chemoautotrophs capable of acquiring energy from reduced iron and sulphur compounds. Samples recovered from the much shallower Lake Whillans in West Antarctica revealed a diverse but recognisable community of bacteria and archaea probably supported by chemolithoautotrophy, closely related to microorganisms able to use reduced nitrogen, iron and sulphur compounds as energy sources. Grzymski et al. [129] described an example of psychrophile detection using six environmental fosmid clones from a library that was created from DNAs collected in Antarctica (nearshore waters off Palmer Peninsula). Each clone represents a different uncultivated marine bacterial group covering the following four phyla: the Gemmatimonadetes, Proteobacteria, Bacteroidetes, and a high-G+C Gram-positive bacteria group.

A singularly cold place is represented by glacier ice in which the temperatures range from −5 °C to −10 °C, and high hydrostatic pressure and low nutrient and poor water availability are present. In this niche, Actinobacteria, Firmicutes, Proteobacteria, CFB (Cytophaga-Flavobacterium-Bacteroides), some fungi and yeasts as well as a few Archaea are present. Simon et al. [130] reported similar taxonomic representation using a metagenomic approach; this is the first metagenomic map on the phylogenetic diversity of glacial ice. This metagenome analysis revealed the potential utilization of a variety of organic substrates and a significant number of genes that are involved in the carbon fixation of autotrophic microorganisms.

### 3.3. Applications

#### 3.3.1. Enzyme Kinetics

Another fundamental question relies on the working mode of the enzymes: at a very low temperature, how can they catalyse all the metabolic reactions that occur in the cell? How can all the processes be ensured? In general, low temperatures imply the decreased functionality of the cells; indeed, the catalytic constant *k_cat_* is exponentially dependent on the temperature. In contrast, most of the psychrophilic enzymes were observed to reduce the reaction activation energy value, increasing the *k_cat_*. In addition, to allow easy substrate-enzyme binding, the active site is generally ampler; there is a lower substrate affinity, with a higher Michaelis-Menten constant [127].

Another strategy by some psychrophilic microorganisms to survive in cold environments, particularly when they are entrapped in ice crystals, consists in the production of antifreeze proteins (AFPs). The first bacteria with documented AFPs were *Micrococcus cryophilus* and *Rhodococcus erythropolis*. *Pseudomonas putida*, a rhizobacterium that was isolated from the Arctic continent and *Marinomonas primoryensis*, isolated from sea ice from Japan, are good producers of AFP, as reported by Kawahara et al. [126] and by Siddiqui [127], respectively (Table 5).

All those listed properties make the psychrophilic enzymes particularly interesting and attractive for applications in industrial production processes [128,131].

They are preferable because, due to their high activity, even small amounts are sufficient for different applications, and their accumulation procedures by means of microbial fermentation are energetically inexpensive because they are performed at low temperatures. Furthermore, high enzyme thermolability stops the process rapidly through only a high temperature gain. In fact, the prospective applications are numerous and involve all the energetic, economic and biotechnological aspects of the production process, both industrial and domestic. The opportunity to run a washing machine efficiently at a lower temperature by using detergents enriched by cold hydrolytic enzymes or to produce fermentable sugars from cellulose, hemicelluloses and starch biomass by employing cold-acting cellulase, xylanase and amylase are in progress. All these procedures would result in inexpensive and eco-friendly products [132,133].

One of the most industrially exploited enzymes is proteases, which are involved in cleaning processes [7]. They are widely used in detergent preparation; therefore, as mentioned above, a working mode at lower temperatures is preferable for energetic reasons, both for industrial and domestic use. Among the several investigated proteases, subtilisins, which are serine proteases that were isolated from an antarctic *Bacillus* species, will act in accordance with all the above reported characteristics. The beta-galactosidase from *Pseudoalteromonas haloplanktis*, which was isolated in Antarctica, has been patented and will be involved in the industrial hydrolysis of lactose to obtain D-tagatose. This latter is a natural sweetener that is a weakly caloric monosaccharide with a low glycaemic index [134]. Several psychrotrophic xylanase enzymes have been studied and evaluated for their potential use in the bread industrial production; when employed as additives, they are able to improve the quality of bread and increase its volume efficiently. At present, one of these xylanases is commercialized by a Belgian company (Table 5) [132].

#### 3.3.2. Exopolysaccharide Producing Psycrophilic Microorganisms

The EPS-producing marine bacterium *Alteromonas macleodii* subsp. *fijiensis* has been isolated from the polychaete annelid *Alvinella pompejana*. Its EPS (HYD657) has been commercialized for cosmetic use to protect sensitive skin against chemical, mechanical and UVB aggressions [25].

From the South Shetland Islands in Antarctica, a cold-adapted bacterium called *Pseudomonas* sp. ID1 was isolated from a marine sediment, and its exopolysaccharide is primarily composed of glucose, galactose and fucose, and it has a molecular mass that is higher than 2 × 10^6^ Da [101,110]. The EPS formed highly stable emulsions and conferred cryoprotection for the producer strain as well as for other tested bacteria, suggesting a universal cryoprotectant role for the food, pharmaceutical and cosmetic fields. The psycrophilic *Pseudomonas* strain CAM025 was isolated from sea ice, and it is responsible for the synthesis of sulphated heteropolysaccharides with high levels of uronic acids and acetyl groups. Since the pH of seawater is 8.0, many of the acidic groups present on this EPS are ionized, allowing for the uptake of dissolved metals (Table 5) [110].

In addition, the tendency to produce polysaccharides is a documented cold adaptation behaviour of various psychrophilic microbial cells. The polymeric substance works as a cryoprotectant by preventing direct contact with the cold environment and therefore allowing the colonization of more extreme niches. The psychrophilic bacterium *Colwellia psychrerythraea* strain 34H produced both an exopolysaccharide and a capsular polysaccharide as reported by Marx et al. [96] and Carillo et al. [97], respectively.

The EPS that was isolated from deep-sea bacterium *Zunongwangia profunda* SMA87 showed antioxidant activity. It could represent a model for developing antioxidant additives for food products, thanks to the prevention of protein and lipid oxidative damage as well as for the cosmetic and medical sectors [135].

## 4. Habitat: Hypersaline Environments

Hypersaline environments are extreme habitats in which the salinity is much higher than that of seawater. Depending on whether they originated from seawater or not, they can be divided into two primary types, that is, thalassohaline and athalassohaline, respectively.

Chemically, thalassohaline environments are characterized by a clear predominance of Cl^−^ and Na^+^ (which are responsible for 49% and 42% of the total molarity, respectively). Other important ions are Mg^+2^, SO_4_^−2^, K^+^, Br^−^, HCO_3_^−^, and F^−^. The average salinity of seawater is 3.5%; when it is concentrated (as in a solar saltern), its composition changes due to serial precipitations. The first precipitates are carbonates, but they form in small amounts. At approximately 10% salinity, calcium carbonate starts to precipitate. The primary precipitation regarding NaCl (halite) takes place at 34% salinity.

The class of extremophilic microorganisms that have specialized in living in extreme hypersaline environments are designated as halophiles. Different authors use different definitions for what constitutes a halophile; the most popular definition of halophiles identifies them as microorganisms that grow optimally at Na^+^ concentrations greater than 0.2 M [136]. According to the optimal salt concentration for growth, these organisms are classified into three categories as follows: extreme halophiles that grow in an environment with 3.4–5.1 M (20% to 30%, *w*/*v*) NaCl; moderate halophiles that grow in an environment with 0.85–3.4 M (3% to 25%, *w*/*v*) NaCl; and slight halophiles that grow in an environment with 0.2–0.85 M (1% to 5%, *w*/*v*) NaCl [137].

### 4.1. Microbial Diversity

Marine salterns are habitats for a large variety of halophilic or halotolerant bacteria that develop throughout the entire salt concentration gradient. Evaporation of hypersaline brines is frequently observed, leading to a gradient of salinity, which in turn leads to sequential blooms of diverse microbial species adapted to different ranges of salinity. In solar salterns, as brine is concentrated from 1 M NaCl to approximately 3.5 M. Purple and green sulfur and non sulfur bacteria cover the bottom of many hypersaline ponds [138]. In the anoxic zones of the mats and in the sediment below, a variety of sulfur oxidising, sulfate reducing, homoacetogenic, methanogenic, and heterotrophic bacteria and archaea occur, including aerobic members of *Archaea* belonging to the genera *Halobacterium*, *Natronobacterium*, *Haloferax* and *Haloarcula* in addition to several species pertaining to the *Bacteria* and *Eukarya*. From approximately 4 M NaCl to saturation (>5.1 M NaCl), halophilic archaea dominate the brine pools and most other microbial activity ceases [137]. Only the methanogenic species of the *Archaea*, *Methanohalobium evestigatum* was reported to grow optimally at NaCl concentrations over 20% [139].

#### 4.1.1. Halophilic Microorganisms

A variety of halophilic bacteria were also isolated from sea sands and seaweeds. Thus, the sea contains many moderately halophilic or at least extremely halotolerant bacteria. In a study of Spanish saltern ponds of intermediate salinity (between 15 and 30% sea salts) (Alicante on the Mediterranean coast, Huelva on the Atlantic coast), the dominant types of colonies that developed on agar plates were assigned by numerical taxonomy to the genera *Salinivibrio*, the *Pseudomonas-Alteromonas-Alcaligenes* group, *Acinetobacter* and *Flavobacterium* (Table 6). They grew optimally in the presence of 10% salts and could be found at salt concentrations of up to approximately 25%. *Salinivibrio* species dominated below 15% salt, while bacteria assigned to the *Pseudomonas*, *Alteromonas*, and *Alcaligenes* groups were especially abundant above 15%. *Flavobacterium* and *Acinetobacter* were found in smaller numbers and were evenly distributed up to 30%, while Gram-positive cocci were found mostly above 25% salt [140].

Most known halophiles are relatively easy to grow, and members of the genera *Halobacterium*, *Haloferax* and *Haloarcula*, for example, have become popular models for studying the archaeal domain because they are much simpler to handle than methanogenic and hyperthermophilic *Archaea.*

Hypersaline environments include hypersaline marine basins where salt deposits dissolve into deep-sea water and create distinct “lakes” of high-density brines on the seafloor of the Red Sea, Mediterranean Sea and Gulf of Mexico [141]. These deep-sea hypersaline anoxic basins (DHABs) have been the recent focus of molecular microbial investigation, because they are interesting environments to search for novel microbes. Apart from their increased high salinity, they are anaerobic and form characteristically sharp brine-seawater interfaces, with some of the brines displaying significant increases in temperature and metal concentration. The ionic composition of the brines generally differs from that of seawater; they are anaerobic, and in some cases the temperature can be elevated as well. The microbiology of Shaban Deep and other deep-sea brines in the Red Sea have yielded a number of interesting microorganisms, including *Salinisphaera shabanensis* (a facultative anaerobe growing in a very large range of salt concentrations, from 1 to 28%) [142], *Flexistipes sinusarabici* (an anaerobe tolerating between 3 to 18% NaCl) (28), and *Haloplasma contractile* (a contractile bacterium, phylogenetically equidistant to the Firmicutes and the Mollicutes), *Halorhabdus tiamatea* (a nonpigmented representative of the Halobacteriales that prefers an anaerobic life style) [143].

Other examples of halophilic microorganisms and their biomolecules are reported in Table 7.

#### 4.1.2. Adaptive Strategies

Halophiles have developed different adaptive strategies to support the osmotic pressure induced by high NaCl concentrations. Some extremely halophilic bacteria accumulate inorganic ions (K^+^, Na^+^, and Cl^−^) in the cytoplasm, which is a type of “salt-in” strategy to balance the osmotic pressure of the environment. Moreover, they have also developed specific proteins that are stable and active in the presence of salts [175,176,177,178]. The stability of the enzymes depends on the negative charge on the surface of the protein due to acidic amino acids, the hydrophobic groups in the presence of high salt concentrations and the hydratation of the protein surface due to the carboxylic groups that are present in aspartic and glutamic acids [179]. In addition, negative surface charges are thought to be important for the solvation of halophilic proteins, to prevent denaturation, aggregation and precipitation.

A strategy of osmotic adaptation is to exclude salts from the cytoplasm as much as possible, and to accumulate organic solutes to provide osmotic balance. A variety of compounds is used for the purpose, ranging from glycerol and other sugar alcohols, amino acids, and derivatives such as glycine betaine and ectoine (2-methyl -1,4,5,6—tetrahydropyrimidine -4-carboxylic acid) and its 5-hydroxy derivative, to simple sugars such as sucrose and trehalose [139].

Another adaptation mechanism that has developed is the lipid composition. Structural adaptations have been observed in the S-layers of halophiles. An extreme halophile contains sulphated glucuronic acid residues and a higher degree of glycosylation, leading to an increased density in surface charges. This characteristic demonstrates an adaptation in response to the higher salt concentrations experienced by *Halobacterium salinarum.* Moreover, in *Haloarchaea*, some S-layer glycoproteins are enriched in acidic residues [180].

### 4.2. Metagenomic Analysis

Ghai et al. [181] described the microbiota of two hypersaline saltern ponds collected from the ponds of Bras del Port salterns, (Alicante, Spain), and they provided a more realistic view of the microbial population with respect to classic culture-dependent methods. The analyses of these metagenomes (nearly 784 Mb) showed the dominance of *Haloquadratum walsbyi* but also revealed novel microbial groups. In fact, the authors described a group with a low GC content in *Actinobacteria* and revealed three new abundant microorganisms, underlining the utility of this approach. They found a low-GC *Euryarchaeon* containing the lowest GC content described for any *Euryarchaeon*, a high-GC *Euryarchaeon* and a *Gammaproteobacterium* related to *Alkalilimnicola* and Nitrococcus. Subsequently, Leon et al. [20] described the isolation and characterization of the most abundant bacterium according to the previous metagenomic studies. This bacterium, which was described as a new genus and new species, was named *Spiribacter salinus*. Metagenomic studies from the Santa Pola saltern of two intermediate-salinity ponds with 13% and 19% NaCl and two crystallizer ponds with 33% and 37% NaCl were recently carried out [6]. The only phyla shared by the four datasets were *Euryarchaeota* and *Bacteroidetes*, with *Euryarchaeota* dominating as salinity increased. The phylum *Bacteroidetes* had similar abundances in each metagenomic dataset (around 7–15%) but generic affiliation was different. Members of the class *Gammaproteobacteria* were abundant in the 13 and 19% salinity datasets. The microbial diversity in these intermediate salinity ponds was larger, containing representatives from 7 to 9 different higher taxa. A deep-executed study by Fernández et al. [182] provided a metagenomic analysis of hypersaline environments, and it underlined the carbon and nitrogen biogeochemical cycling ability of the halophilic population by using light as an energy source via bacteriorhodopsins. In addition, the haloresistance mechanisms of these communities were also reported through the synthesis of compatible solutes such as ectoine, betaine and trehalose, which function as osmoprotectants.

Ceylan et al. [183] studied the osmoadaptive mechanism in *Halomonas* sp. AAD12 and investigated its proteome maps and osmolyte accumulation strategy under salt stress.

Metagenomic studies from halophilic environments have led to the discovery of the “*Nanohaloarchaea*”, a new class of uncultivated microorganisms with a unique amino acid combination in their proteins that is able to increase protein flexibility and osmotic resistance, and they have a very small cell size and atypical archaeal metabolic pathways [184].

### 4.3. Applications of Halophiles

#### 4.3.1. Enzymes

Important biotechnological applications rely on halophilic enzymes. One of the important classes of enzymes produced by halophilic microorganism is represented by hydrolases, such as DNAases, lipases, amylases, gelatinases and proteases, capable of functioning under conditions that lead to precipitation or denaturation of most proteins. Generally, halophilic hydrolases are thermostable and adaptable to a wide range of pH values. The halotolerance of hydrolases derived from halophilic bacteria can be exploited whenever enzymatic transformations are required to function under physical and chemical conditions, such as in the presence of organic solvents and extreme conditions in terms of the temperature and salt content [169]. The commercial use of halophilic hydrolases, e.g., amylases, has been reported for starch degradation, and proteases are used for detergent formulations [185,186].

Singh et al. [187] studied the biodiversity and enzymatic potential of haloalkaliphilic bacteria from saline habitats along coastal Gujarat in India, describing a large number of bacteria that are able to produce proteases, amylases, chitinases and lipases using metagenomic approaches. These enzymes provide a unique model for studying stability and protein folding under extreme conditions, and they displayed salt-dependent resistance as well as organic solvent resistance against denaturation. Also, the halophile organisms contain enzymes that maintain their activity at high salt concentrations, alkaline pH and high temperatures (Table 7) [188].

#### 4.3.2. Biopolymers

The biopolymers are another attractive application because they can be used as emulsifiers, thickeners etc. The halophilic microorganisms can produce liposomes which are used as transporters of compound in medicine, cosmetology and polyhydroxyalkanoates to generate biodegradable polymers, specially halophilic polysaccharides such as sulfated polysaccharides produced from *Halomonas* sp., others with substantial quantity of fucose are produced from *Salipiger mucescens* and have a high potential and value [189]. Halophilic polysaccharides, such as sulphated polysaccharides from *Halomonas* sp. and others with a substantial quantity of fucose from *Salipiger mucescens*, have high potential and value. An interesting levan polysaccharide was produced by *Halomonas smyrniensis* [151], and it had several properties ranging from drug carrier delivery to bioplastic behaviour (Table 7).

#### 4.3.3. Osmolytes

Osmolytes (also known as compatible solutes) that are accumulated or synthesized by extremophilic microorganisms are termed extremolytes. Many halophilic bacteria accumulate ectoine or hydroxyectoine as the predominant compatible solutes. Other types of osmolytes include glycine, betaine and other neutral glycerols [188].

Compatible solutes of halophilic bacteria are used in cosmetics and improving hydration properties generally [190]. Osmolytes can be used for applications in medicine, cosmetology, dermatology and nutrition. In a study that was performed in vitro, ectoine was found to be an effective inhibitor of amyloid formation (protein aggregation from misfolding diseases), which is involved in Alzheimer’s disease and spongiform encephalopathies. In addition, in the latest developments in the dermatology field, ectoine is used in skin care products, and the German company Bitop has introduced a therapeutic cream (Med Ectoin Syxyl) for treating neurodermatitis and psoriasis.

Halophiles have been considered useful objects for ecological and evolutionary studies. One reason for this interest is the need to understand the biochemical mechanisms involved under these extreme conditions because of possible biotechnological use of enzymes and molecules from such organisms [191].

Halophiles produce a large variety of stable and unique biomolecules that may be useful for practical applications. The halophiles have been used for the biodegradation of organic pollutants, the desalinization of wastewater, in nanotechnology, and in producing biopolymers and osmoprotectors (Table 7) [151,180,183].

## 5. Poly-Extremophiles

Microorganisms living in extreme environments utilize a number of adaptive mechanisms in order to enable them to proliferate, and this is true to an even greater extent of poly-extremophiles. Marine extremophiles are the organisms that can thrive and reproduce at extremes of salt concentrations (salinity >1.0 M NaCl), pH (>8.0, <5.0), temperature (1–15 °C, >45 °C), and pressure (average 380 atmosphere, >500–1200 atmosphere and beyond), in the presence of high radiations, recalcitrant compounds, heavy metals, and inhibitors. Extremophiles belonging to the *Eubacteria*, *Archaea*, and *Eukarya* kingdoms produce extremophilic biomass in ecological niches such as oceans, salt marshes, solar salterns, hypersaline lakes, hot springs, marine hydrothermal vents, and soda lakes. These marine polye-xtremophiles have great importance and contributed a lot in biotechnological industries. Bioactive compounds such as extremozymes, proteins, and extremolytes are exploited in various bioprocesses and industries [192,193].

Of all the extremely halophilic archaea, only two species are extremely halophilic, obligately alkaliphilic and thermophilic, and are thus termed poly-extremophiles: *Natrialba hulunbeirensis*, which has a [Na^+^] optimum of 3.4 M, a pH optimum of 9.0, and a temperature optimum of 50 °C [192]; and *Natronolimnobius ‘aegyptiacus’*, which has a [Na^+^] optimum of 4.5, a pH optimum of 9.5 and a temperature optimum of 55 °C [193]. Also of note are *Natronorubrum tibetense*, with a [Na^+^] optimum of 3.4, a pH optimum of 9.0, and a thermotolerant temperature optimum of 45 °C [194] and *Natronorubrum bangense*, with a [Na^+^] optimum of 3.8, a pH optimum of 9.5 and a thermotolerant temperature optimum of 45 °C [194].

The anaerobic extremely halophilic alkalithermophiles, *Natranaerobius thermophilus*, *Natranaerobius trueperi* and *Natronovirga wadinatrunensis*, were isolated from the solar-heated, alkaline, hypersaline lakes of the Wadi An Natrun, Egypt (temperatures up to 60 °C measured in the salt brine) [195,196]. The Wadi An Natrun is a series of eight lakes in northern Egypt noted for their salinity and alkaline pH. *Halorhodospira halochloris*, an anaerobic phototrophic purple bacterium, which is a thermotolerant, rather than thermophilic alkaliphilic halophile, was also isolated from the Wadi An Natrun [197]. *Natranaerobius 'jonesii'*, and the thermotolerant *Natranaerobius'grantii'*, were isolated from sediment samples from Lake Magadi, in the Kenyan Rift Valley [198]. Lake Magadi, like the lakes of the Wadi An Natrun, is noted for its salinity and alkalinity.

The fact that these microorganisms can not only survive but thrive under these multiple extreme conditions has extended the known boundaries for life at a combination of multiple extrema.

Polyextremophilic enzymes have been applied in the food, detergent, chemical, pulp and paper industries. A thermo-alkali-stable enzyme from *Bacillus halodurans* TSEV1 has applicability in pre bleaching of paper pulp and recently has been expressed in *Pichia pastoris* for the production of oligosaccharides [199,200]. Another strain of *B. halodurans* PPKS-2 produced an alkaliphilic, halotolerant, detergent and thermostable mannanase. This strain grows in agro wastes and can be applied for mannanase production on an industrial scale for detergent and pulp and paper bleaching [201]. The Antarctic cold-adapted halophilic archeon *Halorubrum lacusprofundi* produces a recombinant poly-extremophilic enzyme that is active in cold temperatures, high salinity and stable in aqueous-organic mixed solvents. This enzyme is suitable for applications in synthetic chemistry [202]. A thermo-alkali-stable enzyme from a novel poly extremophilic *Amphibacillus* sp. NM-Ra2 was purified and characterized. The enzyme is halophilic, thermophilic, alkali-stable and stable in the presence of different surfactants and organic solvents, and thus has the potential for application in different industries [172].

## 6. Conclusions

**Figure 2:**
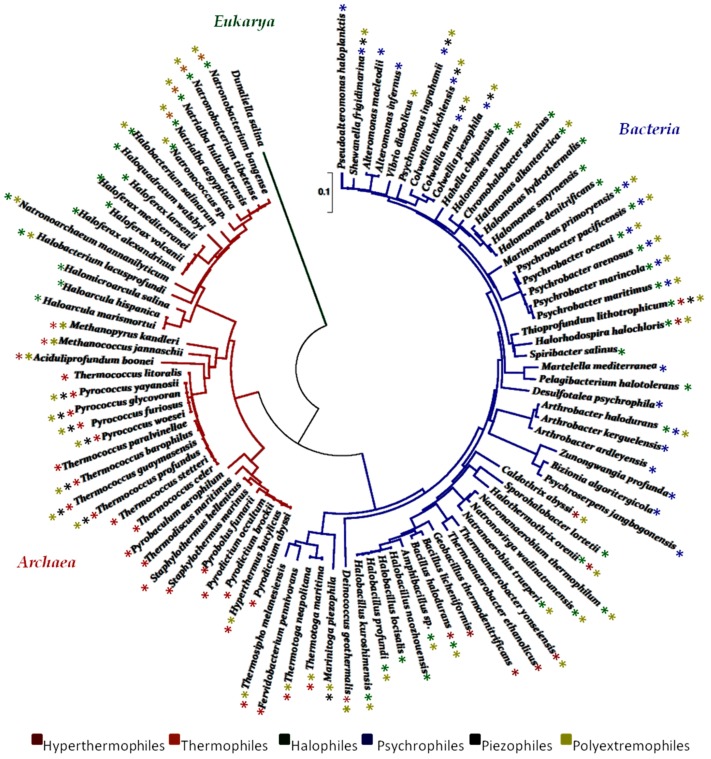
The Neighbor-Joining tree showing the phylogenetic position of representative marine extremophilic species, based on partial 16S rRNA gene sequences and each species identified with the colour code. Bar, 0.1 expected nucleotide substitutions per site. Evolutionary analyses were conducted in MEGA5 [203,204,205].

The biodiversity of ecosystems has become an object of intensive study, leading to a rich body of information regarding the distribution of microbial communities in the world. In recent years, many authors have described the marine microbes of extreme habitats and a representative tree of life of marine extremophiles, based on 16S rRNA sequences, was designed in Figure 2. The isolation of new compounds from such natural sources remains the primary objective together with studies regarding the compounds-biological systems relationship. In particular, enzymes from extreme marine microorganisms are widely used in the chemical industry, and they are also essential for other industries in which they are needed as biological catalysts. Examples range from the production of beer and biofuels to the biological detergents and paper industry. Marine organisms such as bacteria, fungi, sponges and algae have been identified as an unexplored source of enzymes, but they remain somewhat underused. Only a very small part of the marine enzymes has in fact reached the commercialization stage. In addition, the search for high EPS-producing strains is an ongoing process, and the improvement of the fermentation conditions and the subsequent downstream steps for the recovery of the resulting EPS are still in progress. Genetic and metabolic engineering for the production of polymers with well-defined properties as well as the exploration of low-cost substrates for their production are necessary for the widespread use of biopolymers of microbial origins.

Given that most microbes from extreme environments cannot be grown under standard laboratory conditions, new technologies and metagenomics will play a central role in the coming years [206]. The development of new technologies for the discovery of genes through bioinformatics will open many new research avenues.

Microbiological resources from the sea could provide industries with an almost unlimited source of safer products, both economic and ecological, as long as this resource is managed in a responsible manner.

## Figures and Tables

**Table 1:** Basic metabolism of selected hyperthermophilic archaeal species isolated from hydrothermal vent sites. Modified from Canganella [24].

Microorganisms	Growth Metabolism	Site and Temperature of Isolation	Reference
*Pyrodictium occultum*	H₂+ S° ⇒ H₂S	Submarine solfataric field off Vulcano, ItalySSubmarine field off Vulcano, Italy; 105 °C ubmarine solfataric field off Vulcano, Italy;	[30]
*Pyrodictium abyssi*	Organic [H] + S° ⇒ H*₂*S + CO*₂* +organic acids	Marine hot abyssal sites black smokers; 97 °C	[31]
*Hyperthermus butylicus*	Organic [H] + S° ⇒ H*₂*S + butanol + propionic acid	Hydrothermally heated flat-sea sediments off the coast of Sari Miguel, Azores; 100 °C	[32]
*Pyrococcus woesei*	Organic [H] + S° ⇒ H*₂*S	Vulcano Island Beach, Italy; 104 °C	[32]
*Methanopyrus kandleri*	H*₂* + CO*₂* ⇒ CH_4_	“Black smoker” chimney, sea floor of the Gulf of California; 98 °C	[24]
*Pyrolobus fumarii*	H₂ + NH_3_ (or S_2_O_3_)^−2^ ⇒ NH_4_^+1^ (or H_2_S)	“Black smoker”; Mid Atlantic Ridge (depth 3650 m); 106 °C	[26]
*Staphylothermus marinus*	Yeast extract ⇒ H_2_S + CO_2_ + organic acids	Beach of Vulcano, Italy, 92 °C	[26]
*Thermococcus guaymasensis*	Organic [H] + S° ⇒ H_2_S	Guaymas Basin hydrothermal vent, California, 88 °C	[24]
*Thermococcus barophilus*	Organic [H] + S° ⇒ H_2_S	Hydrothermal vent site on the Mid-Atlantic Ridge; 85 °C	[24]
*Thermodiscus maritimus*	Organic [H] + S° ⇒ H_2_S	Hot marine sediment at the beach of Vulcano Island; 90 °C	[26]
*Methanococcus jannaschii*	H_2_ + CO_2_ ⇒ CH_4_	“White smoker” chimney on the East Pacific Rise at 20°50′ N latitude and 109°06′ W longitude at a depth of 2600 m; 88 °C	[26]
*Thermococcus* *paralvinellae*	Organic [H] + S° ⇒ H₂S	Hydrothermal vent chimneys in the north-eastern Pacific Ocean; 28–88 °C	[33]
*Pyrococcus furiosus*	Organic + S° ⇒ H₂CO₂: + H₂S	Geothermally heated marine sediments at the beach of Porto di Levante, Vulcano, Italy; 100 °C	[26]

**Table 2:** Selected examples of thermophilic microorganisms isolated from marine habitats.

Microorganisms	Site and Temperature of Isolation	References
*Bacillus thermodenitrificans* strain B3-72, *Bacillus licheniformis* strain B3-15	Water of a shallow hydrothermal vent, Vulcano Island, Italy	[21]
*Geobacillus* sp. strain 4004	Sediment in marine hot spring near the seashore of Maronti, Ischia Island, Italy	[39]
*Hyperthermus butylicus*	Hydrothermally heated flat-sea sediments off the coast of Sari Miguel, Azores; 107 °C	[32]
*Pyrobaculum aerophilum*	Maronti Beach, Ischia, Italy; 100 °C	[40]
*Thermus aquaticus*	Thermal springs in Yellowstone National Park and thermal spring in California; 70 °C	[41]
*Pyrolobus fumarii*	“Black smoker”; Mid Atlantic Ridge (depth 3650 m); 106 °C	[26]
*Pyrococcus glycovorans*	Deep-sea hydrothermal vent located on the East Pacific Rise; 95 °C	[42]
*Pyrococcus yayanosii*	Deep-sea hydrothermal vent, Mid-Atlantic Ridge; 98 °C	[43]
*Pyrodictium abyssi*	Marine hot abyssal sites black smokers; 97 °C	[31]
*Pyrodictium brockii*	Submarine solfataric field off Vulcano, Italy; Submarine solfataric field of Vulcano Italy, 105 °C	[30]
*Staphylothermus hellenicus*	Geothermally heated vents off Palaeochori Bay, Milos, Greece; 85 °C	[44]
*Thermococcus celer*	Solfataric marine water hole on a beach of Vulcano, Italy; marine solfataric fields of Kraternaya cove (Ushishir archipelago, Northern Kurils 88 °C	[45]
*Thermococcus litoralis*	Shallow submarine thermal spring from Lucrino and Vulcano in Italy; 88 °C	[38]
*Thermococcus stetteri*	Marine Ushishir archipelago, Northern Kurils solfataricus fields of Kraternaya 75 °C	[46]
*Thermococcus profundus*	Deep-sea hydrothermal vent, Middle Okinawa Trough; 80 °C	[47]
*Thermococcus aegaeicus*	Geothermally heated vents off Palaeochori Bay, Milos, Greece; 88–90 °C	[44]
*Thermococcus atlanticus*	Deep-sea hydrothermal vent in the Mid-Atlantic Ridge; 85 °C	[48]
*Thermococcus cleftensis*	Hydrothermal vent chimneys in the north-eastern Pacific Ocean; 88 °C	[33]
*Thermococcus nautili*	Hydrothermal deep-sea vent, East Pacific Rise; 80 °C	[49]
*Thermococcus* *prieurii*	Deep-sea hydrothermal vent, East Pacific Rise; 85 °C	[50]
*Thermococcus thioreducens*	Deep-sea hydrothermal vent site on the Mid-Atlantic Ridge; 83–85 °C	[51]
*Thermotoga maritima*	Geothermally heated locales on the sea floor; 80 °C	[52]

**Table 3:** Selected examples of biomolecules produced from marine thermophilic microorganisms.

Microorganisms	Isolation Sites	Applications	Enzyme	Reference
*Fervidobacterium pennivorans* V5 (recombinant)	Hot springs, Azores islands	Starch conversion; detergent	Amylase debranching	[53]
*Fervidobacterium pennivorans*	Hot spring, Azores island	Poultry industry; detergent; fish industry	Serine peptidase	[53]
*Pyrococcus furiosus*	Hydrothermal vent, Italy	Starch saccharification; detergent	Pullulanase	[45]
*Thermococcus* sp.	Deep-sea hydrothermal vent, USA	Starch conversion; detergent	Alpha-amylase	[45]
*Thermotoga neapolitana* 5068	Hot spring, Italy	Dietary Supplements	Alpha-galactosidase	[54]
*Thermococcus litoralis*	Deep-sea hydrothermal vent, Italy	Detergent	Proline dipeptidase	[55]
*Thermococcus litoralis*	Deep-sea hydrothermal vent Shallow submarine thermal springs and oil wells	Poultry industry; detergent; fish industry	Serine peptidase	[56]
*Thermoanaerobacter yonseiensis*	Geothermal hot stream at Sileri, Indonesia	Poultry industry; detergent; fish industry	Serine protease	[57]
*Aciduliprofundum boonei*	Hydrothermal vent, Mid Atlantic Ridge	Detergent	Cysteine peptidase	[58]
*Caldithrix abyssi*	Deep-sea hydrothermal chimneys, Mid Atlantic Ridge	Detergent	Metallo carboxy-peptidase	[58]
*Deinococcus geothermalis*	Deep-ocean subsurfaces, Italy	Detergent	Thermo-alkali-stable peptidase	[59]
*Salinivibrio* sp. SA-2	Hypersaline brackish water, Iran	Detergent	Lipase	[60]
**Microorganisms**	**EPS-Structure and Chemical Compositions ^1^**		**Applications and Activity**	**Reference**
*Bacillus thermodenitrificans* strain B3-72	*manno-pyranosidic* trisacchacaride repeating unit; Man:Glc Molar ratio:1:0.2		Immunomodulatory and antiviral activities	[21]
*Bacillus licheniformis* strain T4	beta-*manno-pyranosidic* trisaccharide repeating unit; Fruc/Fuc/Glc/GalNAc/Man Molar ratios: 1.0:0.75:0.28:tr:tr		Anti-citotoxicy activity	[61]
*Bacillus licheniformis* strain B3-15	Tetrasaccharide repeating unit; Man *manno-pyranosidic* configuration		Antiviral activity	[21]
*Geobacillus* sp. strain 4004	Gal:Man:GlcN:Ara Molar ratios:1.0:0.8:0.4:02		Pharmaceutical application	[39]
*Thermococcus litoralis*	Man		Biofilm formation	[26]
*Vibrio diabolicus*	GlcNAc and GalNAc Molar ratios:1.0:1.0		Regenerating activity on bone and skin	[62]

^1^ Monosaccharide abbreviations: Glc glucose, Gal galactose, Man mannose, Fruc fructose, Fuc fucose, Ara arabinose, GlcNAc N-acetyl glucosamine, GalNAc N-acetyl galactosamine, tr trace amount.

**Table 4:** Selected examples of psychrophilic microorganisms isolated from marine habitats.

Microorganisms	Site and Temperature of Isolation	References
*Arthrobacter ardleyensi*	Antarctic Ardley Island lake sediment; 25 °C	[89]
*Arthrobacter halodurans*	Sea water collected from the South China Sea; 28 °C	[90]
*Arthrobacter kerguelensis*	Sea water, Kerguelen Islands, Antarctica; 22 °C	[91]
*Arthrobacter subterraneus*	Deep subsurface water of the South Coast of Korea; 28 °C	[92]
*Bizionia algoritergicola*	Sea ice–sea water, East Antarctica; −2 °C	[93]
*Colwellia chukchiensis*	Chukchi Sea in the Arctic Ocean; 23–25 °C	[16]
*Colwellia maris*	Seawater, Abashiri coast off the Okhotsuku Sea, Hokkaido; 0–22 °C	[94]
*Colwellia piezophila*	Deep-sea sediments of the Japan Trench; 10 °C	[95]
*Colwellia psychrerythraea*	Sea ice and marine sediments; Arctic. 8 °C	[16,96,97]
*Pseudoalteromonas haloplanktis*	Antarctic coastal sea water; 12 °C	[98]
*Psychrobacter arenosus*, *P. marincola*, *P. maritimus*, *P. submarinus*, *P. fulvigenes*	Coastal sea ice and sediments of the Sea of Japan; 25–28 °C	[99]
*Psychroserpens jangbogonensis*	Ross Sea in the Southern Ocean, Antarctica; 15 °C	[100]
*Psychrobacter oceani*	Sediment from Pacific Ocean at depth of 7167 m (37°48′ N 143°52′ E); 10–15 °C	[101]
*Psychrobacter okhotskensis*	Monbetsu coast of the Okhotsk Sea in Hokkaido, Japan; 25 °C	[102]
*Psychrobacter pacificensis*	Deep seawater in the Japan Trench off Hachijo Island, Japan; 25 °C	[103]
*Psychromonas ingrahamii*	Sea ice core from Point Barrow, Alaska, USA; −12–10 °C	[104]
*Shewanella frigidimarina* and *S. gelidimarina*	Coastal areas of the Vestfold Hills in eastern Antarctica (68′S 78′′E) Antarctic sea ice; 15–17 °C	[105]

**Table 5:** Selected examples of biomolecules produced from marine psycrophilic microorganisms.

Microorganisms	Isolation Sites	Applications	Enzyme	Reference
*Pseudoalteromonas haloplanktis*	Antarctica	Starch conversion; detergent	Amylase Endo-amylase	[106]
*Zunongwangia profunda*	Deep-sea, China	Starch conversion; detergent	Alpha-amylase	[107]
*Martelella mediterranea*	Lake Martel, Spain	cellulose conversion; detergent	Beta-glucosidase	[22]
*Desulfotalea psychrophila*	Marine sediments, Antarctica	Agriculture; food; pharmaceutical industries	Esterase	[108]
*Pseudoalteromonas haloplanktis*	Marine Antarctic	Detergent	Lipase	[98]
*Psychrobacter* sp. wp37	Deep-sea sediments, West Pacific	Detergent	Lipase	[109]
**Microorganisms**	**EPS-Structure and Chemical Composition ^1^**		**Applications and Activity**	**Reference**
*Pseudomonas* sp. ID1	Carbohydrates 33.8% (Glc 17.0%, Gal 8.6%, Fuc 8.2%)/Uronic acids 2.4%/Proteins 2.8%		Cryo-protection and emulsifying activities	[110]
*Pseudomonas* strain CAM025	Glc/GalA/Rha/Gal Molar ratios: 1:0.5:0.1:0.08		Cryo-protection and trace metal binding	[110]

^1^ Monosaccharide abbreviations: Glc glucose, Gal galactose, Rha rhamnose, Fuc fucose, GalA galacturonic acid.

**Table 6:** Selected examples of halophilic microorganisms isolated from marine habitats.

Microorganisms	Site and Temperature of Isolation	References
*Chromohalobacter salexigens*	Solar saltern, Bonaire, Netherlands Antilles; 37 °C	[144]
*Chromohalobacter salarius*	Solar saltern Cabo de Gata, Almería, southern Spain; 35 °C	[145]
*Halobacterium salinarum*	Badwater salt pan, Death Valley, California; 37 °C	[146]
*Haloferax* *alexandrines*	El-Mallahet, solar saltern near Alexandria City in Egypt; 37 °C	[147]
*Haloferax larsenii*	Solar saltern (122°17’ N 29°55’ E), Zhoushan archipelago, Zhe-Jiang, China; 42–45 °C	[148]
*Halomicroarcula salina*	Yinggehai marine solar saltern near Shanya city of Hainan Province, China; 37 °C	[149]
*Halomonas alkaliantarctica*	Saline lake in Cape Russell, Antarctica; 30 °C	[150]
*Halomonas smyrnensis*	Camaltı Saltern Area, Aegean Region of Turkey, 37 °C	[151]
*Halomonas marina*	Marine water (Hawaii, USA); 37 °C and 26 °C	[152]
*Halomonas pantelleriensis*	Hard sand, lake of Venere, Pantelleria Island, Sicily, Italy; 30 °C	[153]
*Halomonas axialensis*, *H. hydrothermalis*, *H. sulfidaeris*, *H. neptunia*	Hydrothermal fluids low-temperature, sulfide rock and hydrothermal plumes in North and South Pacific Ocean vent fields located at 1530–2580 m depth; 20 °C	[154]
*Halomonas denitrificans*, *H. salaria*, *H. janggokensis*, *H. gomseomensis*	Saline water of Gomseom, solar saltern, saline water of Janggok solar saltern, seawater in Anmyeond; Korea; 25–30 °C	[155]
*Haloarcula marismourti*, *H. hispanica*	Salt lake Dead sea, 31°30′ N, 35°30′ E; 37 °C	[156,157]
*Halobacillus locisalis*	Marine solar saltern, Baekryung Island of the Yellow Sea, Korea; 30 °C	[158]
*Halobacterium halobium*	Solar saltern ,Central-Eastern coast of Tunisia, 34°39′ N and 10°42′ E; 37 °C	[159]
*Halobacteroides halobius*	Salt lake Dead sea sediment ; 37 °C	[160]
*Halobacillus profundi*, *H. kuroshimensis*	Deep-sea carbonate rock at a methane cold seep in Kuroshima Knoll, Japan; 25 °C	[161]
*Halobacillus naozhouensis*	South China Sea, Naozhou Island on the Leizhou Bay; 30 °C	[162]
*Halobacillus seohaensis*, *H. yeomjeoni*	Sediment marine solar saltern, Byunsan, Korea; 37 °C	[163,164]
*Natronoarchaeum mannanilyticum*	Japanese seawater in Niigata prefecture; 37 °C	[165]
*Sporohalobacter lortetii*, *S. marismortui*	Salt lake Dead sea; 35–37 °C	[88]

**Table 7:** Selected examples of biomolecules produced from marine halophilic microorganisms.

Microorganisms	Isolation Sites	Applications	Enzyme	Reference
*Halothermothrix orenii*	Tunisian salt lake	Starch conversion; detergent	Amylase Endoamylase	[166]
*Halomonas sp.* AAD21	Saltern area, Turkey	Starch conversion; detergent	Alpha-amylase	[167]
*Natronococcus sp.*	Soda Lake, China	Detergent	Lipase	[168]
*Pelagibacterium halotolerans*	East China Sea		Metallo beta-lactamase	[169]
*Haloferax volcanii*	Dead Sea, the Great Salt Lake, and oceanic environments with high NaCl	Detergent	Cysteine peptidase	[170]
*Haloferax mediterranei*	Saltern, Spain	Starch conversion; detergent	Endoamylase	[171]
*Amphibacillus* sp. NM-Ra2	Hypersaline alkaline lake Egypt	Starch conversion; detergent	Gluco-amylo pullulanase	[172]
**Microorganisms**	**EPS-Structure and Chemical Composition ^1^**		**Applications and Activity**	**Reference**
*Haloferax mediterranei*	→ 4)-β-D-GlcNAcA-(1→6)-α-D-Man-(1→ 4)-β-D-GlcNAcA-3-*O*-SO_3_^−^-(1→		Candidate in oil recovery overall in oil deposits with high salinity concentrations	[173]
*Halomonas alkaliantarctica* strain CRSS	Glc:Fru:GlcNAc:GalNAc Molar ratio:1.0:0.7:0.3:tr		High viscosity	[11]
*Hahella chejuensis*	Glc:Gal Molar ratio:0.68:1.0		Biosurfactant and detoxification of polluted areas from petrochemical oils	[174]
*Alteromonas infernus*	Repeating unit of uronic acids (GlcA and GalA) and neutral sugars (Gal and Glc) and substituted with one sulfate group		Cartilage tissue engineering applications due to “Heparin-like” behavior	[81]
*Alteromonas macleodii* subsp. *fijiensis*	Glc/Gal/Man/Rha/Fuc/GlcA/GalA Molar ratio: 1/1.9/0.4/0.6/0.2/1.2/2.8		Protection of sensitive skin against chemical, mechanical and UVB aggressions	[25]

^1^ Monosaccharide abbreviations: Glc glucose, Gal galactose, Man mannose, Rha rhamnose, Fruc fructose, Fuc fucose, GlcA glucuronic acid, GalA galacturonic acid, GlcNAc N-acetyl glucosamine, GalNAc N-acetyl galactosamine, tr trace amount.
